# Essentials of Medical Electricity

**Published:** 1892-03

**Authors:** 


ESSENTIALS OF MEDICAL ELECTRICITY. By D. D. Stewart, M. D., Demonstrator of Diseases of the Nervous System, and Chief of the Neurological Clinic in the Jefferson Medical College, etc., etc., and E. S. Lawrence, M. D., Chief of the Electrical Clinic and Assistant Demonstrator of Diseases of the Nervous System in the Jefferson Medical College, etc., etc. With sixty-five illustrations. Philadelphia. W. B. Saunders, 913 Walnut Street. 1892. Price, $1.00.

This is a neat little volume on the Essentials of Medical Electricity, by two distinguished electricians. Everything is practical and to the point. The illustrations are good. It is No. 23 in Saunder's Question Compends.

